# Stem Cell Therapy for Neonatal Hypoxic-Ischemic Encephalopathy

**DOI:** 10.3389/fneur.2014.00147

**Published:** 2014-08-12

**Authors:** Gabriel S. Gonzales-Portillo, Stephanny Reyes, Daniela Aguirre, Mibel M. Pabon, Cesar V. Borlongan

**Affiliations:** ^1^Department of Neurosurgery and Brain Repair, University of South Florida, Tampa, FL, USA

**Keywords:** cerebral palsy, stem cells, hypothermia, combination therapy, translational research

## Abstract

Treatments for neonatal hypoxic-ischemic encephalopathy (HIE) have been limited. The aim of this paper is to offer translational research guidance on stem cell therapy for neonatal HIE by examining clinically relevant animal models, practical stem cell sources, safety and efficacy of endpoint assays, as well as a general understanding of modes of action of this cellular therapy. In order to do so, we discuss the clinical manifestations of HIE, highlighting its overlapping pathologies with stroke and providing insights on the potential of cell therapy currently investigated in stroke, for HIE. To this end, we draw guidance from recommendations outlined in stem cell therapeutics as an emerging paradigm for stroke or STEPS, which have been recently modified to Baby STEPS to cater for the “neonatal” symptoms of HIE. These guidelines recognized that neonatal HIE exhibit distinct disease symptoms from adult stroke in need of an innovative translational approach that facilitates the entry of cell therapy in the clinic. Finally, new information about recent clinical trials and insights into combination therapy are provided with the vision that stem cell therapy may benefit from available treatments, such as hypothermia, already being tested in children diagnosed with HIE.

## Clinical Manifestations of Neonatal Hypoxic-Ischemic Brain Injury

**Figure 1:**
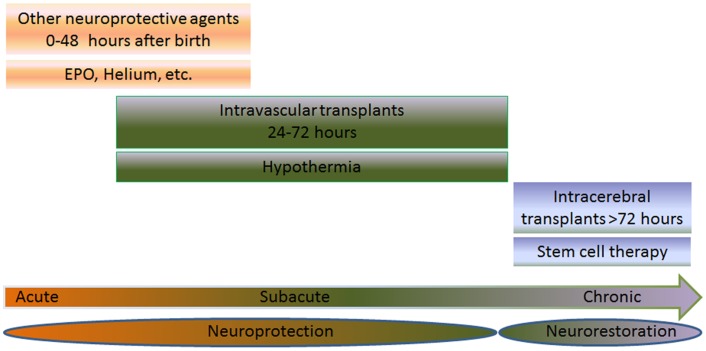
**Multiple intended combination therapies are shown for three different groups: acute (0–48 h after birth), subacute (6–72 h after birth), or chronic (>72 h after birth)**. The joint effort of both cell transplantation therapy and neuroprotective therapies, such as hypothermia, erythropoietin (EPO), and helium are used for treating neonates with HIE. When treating the neonate in the acute or subacute phase, the treatment is referred to as neuroprotection. On the other hand, treatments aimed at the chronic stage are referred to as neurorestoration. EPO or helium is used to treat the subject during the acute phase, while hypothermia and stem cell therapy is used to treat the subacute stage via intravascular routes. When treating the chronic stage, stem cell therapy is used via intracerebral route, which stimulates the neurorestorative mechanisms. The combination of these therapies may prove to be effective in neonates suffering from HIE.

Hypoxic-ischemic encephalopathy (HIE), cerebral palsy (CP), and periventricular leukomalacia (PVL) are mainly triggered by neonatal hypoxic-ischemic brain injury. Neurodevelopmental deficits such as learning disabilities, mental retardation, and hearing and visual impairments accompany children diagnosed with hypoxic-ischemic brain injury. Brain expression of systemic asphyxia characterizes HIE (1). Perinatal asphyxia and resulting hypoxic-ischemic encephalopathy (HIE) occur in 1–3 per 1000 births in the United States (2). Worldwide, 10–60% of infants who develop HIE will die and at least 25% of the survivors will have long-term neurodevelopmental sequelae (2). Hypoxic-ischemic encephalopathy is the primary cause of 15–28% of cerebral palsy among children (2). Throughout the paper, the terms HIE and the alternative term neonatal encephalopathy (NE) (3, 4) are synonymous. These two terminologies have been a topic of much debate (5, 6). Even with an intense effort by researchers and clinicians to employ precise diagnostic methods, encephalopathy has not been identified in premature infants as compared to full term infants (7–9). HIE brings a relatively high 50% mortality rate in newborns (10), and a small portion of those survivors, 25% display CP symptoms permanently (11, 12). Ischemic perinatal stroke is responsible for 30% of children with CP (13). A cerebral white matter injury, known as PVL, is detected in 50% of neonates with exceedingly low birth weights with 90% of survivors displaying CP symptoms (14); however, studies using ultrasonography report findings of the incidence of PVL to be lower than 50% (15–17). As a result of the very similar pathophysiological symptoms between neonatal hypoxic-ischemic brain injury and adult stroke, innovative treatments such as cell-based therapies, which are currently being tested in stroke, may prove to be successful in neonatal hypoxic-ischemic brain injury. Having a grasp of the neurochemical cascade of events is a holy grail for commencing treatment intervention in neonates (18). To this end, therapeutic benefits may be achieved by abrogating the “secondary energy failure” or “excite-oxidative cascade” (18, 19). This is characterized by amplified excitation of NMDA receptors combined with peculiar oxidative stress due to mitochondrial dysfunction, altogether depleting energy from the brain seen in infants with hypoxic-ischemic injury (18). Currently, hypothermia is used to treat HIE (20–22) and has demonstrated to be very effective in newborns with a gestational age of ≥36 weeks (22, 23) diagnosed with moderate to severe HIE (21, 22), but neurodevelopmental deficits persist in 40–50% of patients even after hypothermia (22). A treatment that combines both hypothermia and cell transplantation may prove to be more effective and benefit neonates with moderate to severe HIE (Figure 1).

## Key Preclinical Gating Items for Stem Cell Therapy for HIE

Academicians, industry partners, and regulators, which include both the National Institutes of Health (NIH) and the U.S. Food and Drug Administration (FDA), have jointly created Stem cell Therapeutics as an Emerging Paradigm for Stroke (STEPS). Together, they have provided guidelines to increase the successful outcome of cell therapy in stroke patients (24–31). The need for the establishment of a Baby STEPS consortium is necessary for younger patients (32). This would allow a safe and effective translation of cell therapy in neonatal hypoxic-ischemic brain injury. Below, we identify critical gating criteria in conducting translational studies in order to aid and advance the formation of Baby STEPS guidelines.

### Clinically relevant models of HIE

The animal species and strain should be specially considered in HIE modeling. Rodent models such as Vannucci’s model of neonatal hypoxic-ischemic brain injury parallel many pathological events that humans endure during neonate HIE (33). Researchers found that in 7-day-old postnatal rats, which undertook ligation of unilateral carotid artery as well as systemic hypoxia, suffered widespread cell death to cerebral cortex, subcortical and periventricular white matter, striatum, and hippocampus ipsilateral to the ligated artery (34). Rats are not the only species used to create the Vannucci model, but mice have also been used (35) with varying pathological outcomes dependent on the mouse strain (36–39).

Another equally important variable that should be controlled in the experimental HIE is the age of the animals. Younger animals have shown to be impervious against hypoxia. A 1–2-day-old postnatal rat needs to be exposed to more severe hypoxia as compared to its 7-day-old counterpart in order to attain efficacious HIE symptoms (40). Another important fact to note is that younger animals experience worse white matter injury than older rats (35). Therefore in HIE modeling, age is a critical factor and is further verified by a focal subcortical cell loss paired with a surge in proliferating oligodendrocyte progenitor cells following HIE in young neonates, but rather modest in older models (41–45). Age-related changes following HIE need to become standardized in order to better evaluate the therapeutic benefits of experimental treatments.

Gender should also be taken into consideration for models, in that HIE-induced female neonates displayed a much smaller infarct volume and improved sensorimotor task than their male counterparts after perinatal hypoxic-ischemic brain injury and treatment with erythropoietin (46). A possible explanation behind the differences between female and male neonatal infarct sizes and improved neurological behavior has become increasingly clear. It is known that gender differences in injury to the brain are not merely a result of hormonal influence (47), but the properties of individual cells (48). For instance, male and female cells display differential gene expression even when no hormonal influences are apparent (49), and brain cells show phenotypic differences that are gender dependent but independent of gonadal phenotype (50). Moreover, gender modulates responsiveness to recombinant erythroprotein (Epo) (51). Additionally, Epo receptor (EpoR) alleles, EpoRA1, and EpoRA10, have displayed a significantly higher frequency in females when compared to males (52). Epo administration is known to produce significant long-term neuroprotective benefit on the developing brain (46). This suggests that Epo has a gender preference with neonatal benefit in females, whose mechanisms must be further investigated. Together with other studies demonstrating that gender similarly affects injury models (53, 54), these studies suggest that gender should be carefully considered in experimental HIE.

The approximation of the clinical pathology of HIE is a crucial goal in standardizing the animal models because a model that better mimics a human condition would allow for better evaluations of possible treatments in human patients. Rats along with many other species, which include non-human primate, sheep, lamb, puppy, piglet, and rabbit, have been employed as models to closely resemble some HIE pathological aspects in humans (33, 55–60). Unfortunately, because of the expense in using large animal models, it has deterred research with these clinically relevant models. The piglet model is a good model for research regarding treatment plans for neonates, as it closely resembles the weight and size of a newborn infant. This piglet model also reveals new treatment variables, in that phosphorylated metabolites are temperature-sensitive and that the more severe the energy depletion the worse the secondary energy failure, exacerbating neuronal death (61, 62). These findings suggest the need to control temperature and maintain brain injury in experimental models using therapeutic strategies.

### Clinically relevant experimental paradigms

Lab-to-clinic translatable functional tests need to be developed to better assess the pathological improvements of experimental interventions for short and long-term outcomes that are species specific and mimic the human condition. Despite efforts of investigators to control many variables in experimental HIE models, characterizing the phenotype of encephalopathy in neonates has proven to be elusive. As opposed to adults, neonatal encephalopathy is prevented from implementation of timely interventions as there is a scarcity in studies determining the optimal supra-acute to chronic therapeutic window in laboratory models, thus presenting a major barrier to translating experimental treatments to clinical applications. Acknowledging this research gap is pivotal when designing therapeutic intervention studies for future clinical applications in neonates. In order to create well-designed translational studies, it is critical to optimize the dosage, delivery route, and timing of stem cell transplantation within applicable clinical parameters. Treating the laboratory as the clinical setting for cell therapy in HIE will enhance the translational potential of the stem cell product. In order to prevent any potential microembolism, the minimum therapeutic cell dosage must be determined. Finding minimally invasive procedures for cell delivery could prevent exacerbation of the already injured brain. For timing of cell delivery, consideration should be given to the neuroprotective phase (< 1 day of injury) and the neurorestorative phase (> 1 day after injury) (63, 64). While most transplant studies have examined a single bolus injection of stem cells, we recognize that HIE is a disorder that involves progressive neuronal cell loss even days after HI. As a result, multiple treatments of stem cells and adjunct neuroprotective therapies are more likely to have not only neuroprotective but also neurorestorative effects. This would provide a continual targeted regime to prevent cell death in the hopes to improve neurodevelopmental outcome. For example, two MSC injections at 3 and 10 days after neonatal hypoxia-ischemia (HI) markedly improved sensorimotor function 4 weeks after the insult (65). This MSC-3 + 10 treatment was more powerful in improving functional outcome and in reducing gray and white matter loss than a single MSC injection at 3 days after HI (65).

### Well-defined phenotypic markers of donor stem cells

A detailed description of the distinguishable phenotypic features of stem cells is an integral component of understanding the biology and potential of stem cells (66–74). To determine the safety and effectiveness of stem cells that are to be transplanted in HIE, it is important to know their identity. This will also give way to understanding the mechanisms allowing the functional amelioration that takes place after transplantation. That different cell types in the brain undergo degeneration has prompted the notion that brain repair consists of several mechanisms facilitating various types of cells working together with the trophic, neurogenic, vasculogenic, and other by-stander effects of the transplanted stem cells (75). Accordingly, such multi-pronged cellular repair process necessitates the need to determine the source of the stem cells to aid in realizing the therapeutic effects and mechanisms of action associated with cell therapy. Another aspect to consider is the ability of the cells to be shipped frozen and thawed for transplantation at the clinic, which is important for neonatal diseases which may benefit from early intervention. Moreover, using autologous stem cells can be of an advantage due to their ability to bypass graft rejection along with its side effects. For example, in a study where intravenous injection of autologous cord blood was made, the results showed that they were safe in CP children (76). In parallel, placental tissue obtained during prenatal chorionic villous sampling or at delivery can be a good source of autologous stem cells which can be transplanted during the last month of gestation or the first few months after delivery if neurodegeneration is detected in the baby (77).

### Functional outcomes in cell transplantation in HIE

To assess the safety and efficacy of the treatment group in HIE, specific behavioral and histological procedures are frequently used. These assays can determine motor and cognitive amelioration as well as provide insight to the biochemical processes that are undergoing in the brain (68, 78, 79). However, to value the functional improvement after cell transplantation in animals, a close estimate of the HIE clinical symptoms needs to be shown in these preclinical models (80–83). In addition, monitoring the safety and efficacy of stem cells over time will allow a better assessment of the effects of cell therapy (84). This becomes an obstacle for neonatal HIE due to the spontaneous amelioration seen in the development and maturation phases of the neonatal animal (85) and in pediatric patients (86). More sensitive functional outcomes may be needed to delineate spontaneous recovery from true therapeutic benefits of cell therapy; for example, to examine the grafted cells and host HIE microscopically, specific markers for the trophic factor effect, immunomodulatory response, neurogenesis, vasculogenesis, angiogenesis, and synaptogenesis, as well as inflammation, tumorigenesis, or ectopic tissue formation may be used (87–89). This approach allows a better understanding of the cells’ mechanism of action and indication of unfavorable side effects.

### Mechanisms of action underlying cell therapy

Two major modes of action are involved in stem cell-mediated functional recovery in ischemic brain injury: cell replacement and by-stander effect. Cellular and molecular neurorestorative mechanisms include neurogenesis, angiogenesis, synaptogenesis, immunomodulation, and trophic factor secretion (30, 31, 90). Real-time visualization techniques (i.e., magnetic resonance imaging), originally performed in stroke and extended to HIE models (91–93), have allowed, recently, the tracking of the transplant, as well as the imaging of the host neurorestorative mechanisms (94–100). The neurorestorative mechanism is characterized by transplantation of stem cells that serve as biobridge for the initiation of endogenous repair mechanisms (101). The transplanted stem cells pave the way between the neurogenic niche and injured brain site in order to traffic the migration of host neurogenic cells (101).

## Outstanding Preclinical Issues Necessary for Clinical Trials of Cell Therapy in HIE

Preclinical trials on stem cells in cerebral palsy have been conducted and have shown significant improvement in animal models. However, results have been difficult to replicate in humans since most of the studies have been on acute hypoxic injury models, which are less similar to human cases compared to chronic models. So far, clinical trials on children with cerebral palsy (CP) have suggested that neural progenitor cells (NPC), umbilical cord mononuclear cells, and mesenchymal stem cells (MSC) transplants are safe, and could improve motor and cognitive functions (102). Currently, there are three ongoing registered clinical trials on stem cells and CP on children, and two that have recently been completed.

Two of these clinical trials are currently being conducted in the United States, with the aim of testing the safety and efficacy of autologous cord blood cells. The primary outcomes should be expected by the year 2015. Although patient recruitment is not open, China intends to test the efficacy of umbilical cord MSC versus rehabilitation treatments on children with cerebral palsy. In Iran, two clinical trials have already been completed. These clinical trials studied the efficacy of multiple intrathecal bone marrow-derived CD133 cell transplantation in children with cerebral palsy. Results are not published yet (103).

It is imperative to set the parameters defining safety and efficacy of stem cell therapy in neonatal HIE clinical trials. In the United States, the Medical College of Georgia and Duke University are assessing the safety and efficacy of umbilical cord blood transplants in CP pediatric patients. The long-term efficacy of intravenous transplantation of autologous cord blood in CP children remains to be determined (76, 104). So far, autologous bone marrow-derived MSCs have also been found to be safe when transplanted, but it was only on one CP patient (105). Autologous stem cell sources have been preferred largely due to their safety profile, but cell types that have already committed to a particular lineage or niche in the brain also have the potential to be used as donor cells. In order to implement cell therapy in neonatal ischemic-injury patients, there should be sufficient evidence of safety, efficacy, and mechanism of action of the stem cells in animal models. Moreover, it has been difficult to obtain a projection of the neurologic outcomes of cell therapy in neonatal hypoxic-ischemic injury. The current reports on clinical improvement after cell therapy in children with CP or HIE should not interfere with the need for sufficient preclinical studies for the advancement of clinical trials. The guidelines mentioned in the previous sections on the Baby STEPS may also be implemented to other potential therapies for neonatal hypoxic-ischemic injury (106–109) and should be used along with the current pediatric stroke recommendations in research and treatment interventions (110–113). In the end, while autologous stem cells have shown to be safe and effective as a possible treatment for HIE, more preclinical studies, paralleled by carefully designed limited clinical trials should be conducted before moving into larger studies.

## Cell Therapy and Adjunctive Treatment with Hypothermia

The current therapies for HIE attempt to interrupt the pathways activated by HIE. In neonates with HIE, the results have not been promising in regards to preventing the neuronal loss (114, 115). In research models of HIE, it has been shown that hypothermia reduces the release of glutamate (116), reduces the secondary energy failure (21, 61, 116–118), normalizes protein synthesis (119), and reduces the injury by free radicals (115). Small trials of hypothermia conducted on human neonates (117, 118) showed promising results, while three large trials showed improvement in the neurodevelopment in neonates with mild to moderate HIE, but showed no improvement in neonates with severe HIE (20–22). There is evidence that hypothermia may exert neuroprotection by reducing the neurodevelopmental disability at 18 months of age in newborns with either moderate or severe HIE (120). Neuroprotective approaches could reduce the initial tissue damage, however, cell therapy will still be required to repair the already damaged regions of the brain. This approach can then be implemented on neonates with moderate to severe HIE.

In normal physiological conditions, the endoplasmic reticulum (ER) and mitochondria sequester calcium when intracellular levels increase (121). Calcium enters the cell through voltage-sensitive calcium channels and agonist-operated calcium channels, which are activated by glutamate, *N*-methyl-d-aspartate (NMDA) and kainate, and quisqualate (K/Q) (121). In the ischemic cascade, the increase in hydrogen displaces calcium from intracellular proteins and intracellular calcium levels increase eventually leading to mitochondrial dysfunction (121). In addition, calcium activates intracellular proteases and depolarization occurs in the cell membrane, releasing a large number of excitatory neurotransmitters such as glutamate (121). This activates NMDA receptors persistently, causing intracellular hyperosmolarity in the postsynaptic cell eventually leading to neuronal death (121). Moreover, the ongoing sodium influx inhibits the normal magnesium blockade on NMDA receptors (121). Hypothermia significantly reduces extracellular levels of excitatory neurotransmitters (121). The release of these neurotransmitters is temperature dependent and even mild levels of hypothermia exert an inhibitory effect (121). Hypothermia promotes the survival of neurons through an interaction on glycine since NMDA receptors require the presence of glycine to be activated (121). Hypothermia significantly decreases brain glycine levels after ischemia, thus decreasing hyperexcitability by glutamate (121).

Hypothermia exerts neuroprotection in HIE against aberrant stages of region-specific brain maturation (122), blood–brain barrier (BBB) impairment (123), and apoptosis due to mitochondrial dysfunction (124). Because hypothermia is most efficacious within the first 6 h of life for the infant with moderate to severe HIE (125–127), the patient may benefit even more with the use of a combinatorial therapies (128, 129). For example, using erythropoietin and helium, both which are in clinical trials (130–132), should be considered for in combination therapy. The treatment considerations for hypothermia in addition to combining other therapies are based on the evolving pathophysiology of neonatal brain injury, discussed by Ferriero et al. (133, 134). Using a combination of therapies may be more beneficial to tackle the activated cell death pathways; moreover, detecting it early in at-risk newborns may help prevent or reduce the disabilities following neonatal brain injury (133, 134).

As discussed above, accumulating experimental data have indicated the mobilization of bone marrow-derived stem cells, such as MSCs, in brain plasticity and therapy of HIE to the affected area (135). In the clinic, MSCs can be obtained from umbilical cord blood, adipose tissue, amniotic fluid/tissue, or menstrual blood (136). As alluded earlier, autologous MSCs may be the preferred stem cells to avoid adverse effects associated with graft rejection, but allogeneic MSCs may also be equally safe and effective because of their immature immune system, as well as their capacity to secrete anti-inflammatory factor (136). MSCs are capable of differentiation into variety of phenotype cells (130, 137) and have been demonstrated to exert a therapeutic benefit against brain injury (125). However, little is known regarding MSC treatment for HIE, especially in combination with hypothermia.

The observation that seizure onset beyond the first 12 h of life is not only common in newborns with HIE (138), but also is associated with severe brain injury (56), advances the notion of a critical relationship between the onset of neonatal seizure and initiation of the therapy. Accordingly, any treatment regimen, including hypothermia, is likely to exert benefit if initiated within 6 h after hypoxic-ischemic injury and continuing over the next 12 h or even beyond (i.e., for 72 h) (138). The mechanism underlying hypothermia remains elusive, but may include its capacity to reduce oxidative stress, energy deficit, and inflammation (139). Because of the dismal prognosis of infants with HIE, clinical enthusiasm for a novel treatment is understandable (140). Based on preclinical studies, accumulating evidence suggests that in order to treat more effectively neonatal HIE, and many other neurodegenerative diseases, combination therapy can be of great help. As ischemic brain injuries have two separate cascades of events, one immediately after injury and one persisting even weeks after, it is important to use combination therapy which can tackle both events at different times. We highlight here that hypothermia could be a great neuroprotective method implemented early in HIE, while stem cells could have a better neurorestorative approach, especially during the chronic stage of the disease, which starts days after (i.e., 72 h after birth).

As mentioned previously, combination therapy may be the best approach to treat neonatal HIE, especially when a definite therapeutic time frame has not been fully established. The use of other possible neuroprotective strategies has been studied and is believed to enhance the therapeutic effects of hypothermia by targeting different therapeutic windows and stages of HIE. Oxygen free radicals are usually elevated after hypoxic-ischemic injury. The use of antioxidants like superoxide dismutase, combined with polyethylene glycol to facilitate infiltration across the blood–brain barrier, can degrade reactive oxygen species to ameliorate the negative effects of hypoxia. Nonetheless, neuroprotection in newborn animals has only been seen when administered prior to injury. Xanthine oxidase inhibitors, like allopurinol and oxypurinol, have also shown to reduce concentrations of free radicals in infants when administered early during the recovery phase, while administration of lazeroids appears to inhibit iron-dependent lipid peroxidation in immature models of hypoxic-ischemic brain damage (141, 142). Epo appears to have a longer therapeutic window (24–48 h after delivery) compared to hypothermia. Administration of Epo leads to a reduction in white matter injury, free radical production, and glutamate cytotoxicity in neonates after a hypoxic event by increasing the system XC-expression (130, 142, 143). System XC exchanges 1 mol of cystine for 1 mol of glutamate leading to increased cellular viability (143). The therapeutic effects of hypothermia and IV melatonin in perinatal asphyxia piglets model have been reported (144). Glutamate antagonists such as MK-801 have been show to reduce brain damage after hypoxic-ischemia in neonatal animal models (141, 144). The accumulation of calcium in the cytosol is also characteristic of hypoxic-ischemia. Flunarizine, a Ca + channel blocker, has shown to have a neuroprotective effects when administered prior to injury in fetus and newborn animals (141). Magnesium sulfate has also shown to be a potential treatment to reduce newborn brain injury (in rats) by blocking the neuronal influx of Ca + within the ion channel, while it could also reduce the incidence of moderate to severe CP (141, 144). However, the use of Ca + blockers has been linked to adverse cardiovascular effects on infants, contraindicating their use. It has been observed that neuronal loss can be reduced through the administration of NOS inhibitors in immature rats; however, further studies need to be conducted in order to prove its effectiveness in other animal models (141). Neuroprotective effects after the administration of noble gases like xenon, argon, and helium 2 h post hypoxic injury in 7-day-old SD rat has been reported. Although helium seems to be neuroprotective only in mild hypoxic-ischemic injury, argon and xenon showed neuroprotection in severe hypoxic animal models by increasing Bcl-2 and Bcl-xL, as well as reduction of infarct size and long-term neurocognition (144, 145). There are still many potential neuroprotective strategies to be studied including protection by neural stem cells and the ependymal lining of the ventricles, among others, which will similarly require further investigations for HIE applications.

The use of delta opioid agonists may resemble certain physiological correlation of hibernation, including hypothermia (71), which may involve direct opioid receptor activation, as well as non-opioid mechanisms (71, 146, 147). Interestingly, delta opioids may regulate neural stem and progenitor cell proliferation and differentiation (148), and may even enhance cell-based therapeutics in *in vitro* and *in vivo* disease models (149). Our recent study (150) revealed that moderate hypothermia is efficacious in an *in vitro* model of hypoxic-ischemic injury, which was enhanced by MSC treatment. We also showed that the delta opioid system, along with other non-opioid neuroprotective processes, primarily contributes to the observed neuroprotection in HIE. Stem cell therapy using MSCs significantly improved the therapeutic outcome of moderate hypothermia. Primary rat neurons were exposed to oxygen-glucose deprivation (OGD) condition, a model of hypoxic-ischemic injury, then incubated at 25°C (severe hypothermia), 34°C (moderate hypothermia), and 37°C (normothermia) with or without subsequent co-culture with MSCs. Combination treatment of moderate hypothermia and MSCs proved to be the optimal condition for preserving cell survival and mitochondrial activity after OGD exposure. Pharmacologic induction of hypothermia in human embryonic kidney cells (HEK293) via treatment with delta opioid peptide (DADLE) resembled moderate hypothermia’s attenuation of OGD-mediated cell alterations, which were much more pronounced in HEK293 cells overexpressing the delta opioid receptor. Further, the addition of DADLE to 34°C hypothermia and stem cell treatment in primary rat neurons showed synergistic neuroprotective effects against OGD which were significantly more robust than the dual combination of moderate hypothermia and MSCs, and were significantly reduced, but not completely abolished, by the opioid receptor antagonist naltrexone altogether implicating a ligand–receptor mechanism of neuroprotection. Investigations into other therapeutic signaling pathways revealed growth factor upregulation (i.e., GDNF) and anti-apoptotic function accompanying the observed therapeutic benefits. These results support combination therapy of hypothermia and stem cells for hypoxic-ischemic injury, which may have direct impact on current clinical trials using stand-alone hypothermia or stem cells for treating neonatal hypoxic-ischemic brain injury.

The use of hypothermia as a treatment strategy is not limited to neonates. In adults, hypothermia has been regarded as a therapeutic strategy to improve the patient’s survival and reduce neurologic injury after cardiac arrest (151). Small clinical trials provide evidence of hypothermia as a potential treatment for traumatic brain injury (TBI) as it significantly reduced rates of death, vegetative state, and long-term disability (152). Although not regarded as a treatment strategy for spinal cord injury (SCI), the control of pressure around the injured spinal cord along with the improved behavioral outcome in animal studies demonstrates the potential of systemic hypothermia as a method of treating acute SCI (153). When used in combination with recombinant tissue plasminogen activator, animal data also show a reduction in brain hemorrhage and blood–brain barrier disruption, indicating the synergistic potential of hypothermia in stroke (154).

In addition, the use of stem cells as a therapeutic strategy in adults has obtained much attention due to the substantial beneficial data gathered in animal and clinical studies. For example, specific disease-relevant neurons can be obtained from induced pluripotent stem cells including dopaminergic neurons for Parkinson’s disease (PD), hippocampal and cholinergic neurons for Alzheimer’s disease, motor neurons for amyotrophic lateral sclerosis, and inhibitory interneurons for schizophrenia (155). Embryonic stem cells can be made to also differentiate into dopaminergic neurons and have been shown to ameliorate the behavioral deficit in animal models of PD (156). Embryonic neural stem cells are also considered in demyelinating diseases, such as multiple sclerosis, to replace glial cells that have been lost (156). In addition, the use of neural stem cells in Huntington’s disease has been evident (156).

## Synopsis

Stem cell therapy has the potential to become a treatment method for neonatal hypoxic-ischemic brain injury, but it needs to be first implemented in the clinic on patients with neonatal hypoxic-ischemic brain injury. This will require further translational research studies so that stem cell therapy can be fully implemented. Stem cell therapy can also benefit from the ongoing trials of stem cell therapy in adult stroke due to the similarities in pathology. Neonatal hypoxic-ischemic injury, however, has distinct symptoms from adult stroke (157–159) that will demand modifications to the translational approach before it can be applied to the clinic. Combining both hypothermia and stem cell therapy may further improve the results of cell therapy in neonatal hypoxic-ischemic injury. The results from using this combinatorial approach can be then measured by behavioral and histological assays. In addition, using biomarkers to monitor the transplanted cells in the patient over time will contribute to exposing the long-term effects of this combinatorial therapy.

Currently, the use of stem cells for neonatal hypoxic-ischemic brain injury is still in its experimental phase. Although clinical trials are scheduled to be conducted using autologous umbilical cord blood cells on CP children, there still needs to be a sufficient amount of data demonstrating the safety and efficacy before transplantation therapy can be used in other neonatal diseases. It is a priority to obtain standardized experimental models with quantitative functional endpoints and predictive clinical outcomes so that translational research can be implemented. In addition, it is important to address and modify the following for an effective future use of stem cell therapy: the route of delivery, cell dose, and timing of transplantation after diagnosis of neonatal brain injury. It should also be noted that because it is difficult to detect encephalopathy in premature babies due to the lack of advanced imaging devices and sufficient studies, initial cell therapy clinical trials will consist of full term infants. Because it is agreed that HIE involves several cell death pathways, it is expected that more laboratory research on combination therapies will be conducted in the future. Specifically, incorporating clinical stage therapies, such as hypothermia and other neuroprotective strategies, with stem cell transplantation therapy will be of future focus. Moreover, the safety and efficacy of these combinatorial strategies for neonatal hypoxic-ischemic brain injury can be maximized by following pertinent translational research guidelines [e.g., Ref. (32)].

## Conflict of Interest Statement

Cesar V. Borlongan holds patents and has pending patent applications in stem cell biology and therapeutic applications.

## Abbreviations

CP, cerebral palsy; HI, hypoxic-ischemia; HIE, hypoxic-ischemic encephalopathy; MSCs, mesenchymal stem cells; NE, neonatal encephalopathy; PVL, periventricular leukomalacia; STEPS, stem cell therapeutics as an emerging paradigm for stroke.

## References

[1] Vannucci R Hypoxia Ischemia: Pathogenesis and Neuropathology. St. Louis, MO: Mosby (1997).

[2] Selway LD State of the science: hypoxic ischemic encephalopathy and hypothermic intervention for neonates. Adc Neonatal Care (2010) 10:60–6 10.1097/ANC.0b013e3181d54b30 20386369

[3] Nelson KB Neonatal encephalopathy: etiology and outcome. Dev Med Child Neurol (2005) 47:292 10.1017/S0012162205000563 15892369

[4] Nelson KB Leviton A Hypothermia for neonates with hypoxic-ischemic encephalopathy. N Engl J Med (2006) 354:1643–5 10.1056/NEJMc053092 16615186

[5] Dammann O Ferriero D Gressens P Neonatal encephalopathy or hypoxic-ischemic encephalopathy? Appropriate terminology matters. Pediatr Res (2011) 70:1–2 10.1203/PDR.0b013e318223f38d 21654279

[6] Leviton A Why the term neonatal encephalopathy should be preferred over neonatal hypoxic-ischemic encephalopathy. Am J Obstet Gynecol (2012) 208:176–80 10.1016/j.ajog.2012.07.020 22901708

[7] Blume HK Loch CM Li CI Neonatal encephalopathy and socioeconomic status: population-based case-control study. Arch Pediatr Adolesc Med (2007) 161:663–8 10.1001/archpedi.161.7.663 17606829

[8] De Vries LS Van Haastert IC Benders MJ Groenendaal F Myth: cerebral palsy cannot be predicted by neonatal brain imaging. Semin Fetal Neonatal Med (2011) 16:279–87 10.1016/j.siny.2011.04.004 21636334

[9] Volpe JJ The encephalopathy of prematurity – brain injury and impaired brain development inextricably intertwined. Semin Pediatr Neurol (2009) 16:167–78 10.1016/j.spen.2009.09.005 19945651 PMC2799246

[10] Macdonald HM Mulligan JC Allen AC Taylor PM Neonatal asphyxia. I. Relationship of obstetric and neonatal complications to neonatal mortality in 38,405 consecutive deliveries. J Pediatr (1980) 96:898–902 10.1016/S0022-3476(80)80574-9 7365599

[11] Finer NN Robertson CM Richards RT Pinnell LE Peters KL Hypoxic-ischemic encephalopathy in term neonates: perinatal factors and outcome. J Pediatr (1981) 98:112–7 10.1016/S0022-3476(81)80555-0 7452386

[12] Robertson CM Finer NN Grace MG School performance of survivors of neonatal encephalopathy associated with birth asphyxia at term. J Pediatr (1989) 114:753–60 10.1016/S0022-3476(89)80132-5 2469789

[13] Raju TN Ischemic perinatal stroke: challenge and opportunities. Int J Stroke (2008) 3:169–72 10.1111/j.1747-4949.2008.00205.x 18705894

[14] Khwaja O Volpe JJ Pathogenesis of cerebral white matter injury of prematurity. Arch Dis Child Fetal Neonatal Ed (2008) 93:F153–61 10.1136/adc.2006.108837 18296574 PMC2569152

[15] Chen HJ Wei KL Yao YJ Multicenter investigation for incidence of periventricular leukomalacia in premature infants in China. Zhongguo Dang Dai Er Ke Za Zhi (2008) 10:686–92 19102830

[16] Baud O D’Allest AM Lacaze-Masmonteil T Zupan V Nedelcoux H Delaveaucoupet J The early diagnosis of periventricular leukomalacia in premature infants with positive rolandic sharp waves on serial electroencephalography. J Pediatr (1998) 132:813–7 10.1016/S0022-3476(98)70309-9 9602191

[17] Olsen P Paakko E Vainionpaa L Pyhtinen J Jarvelin MR Magnetic resonance imaging of periventricular leukomalacia and its clinical correlation in children. Ann Neurol (1997) 41:754–61 10.1002/ana.410410611 9189036

[18] Johnston MV Fatemi A Wilson MA Northington F Treatment advances in neonatal neuroprotection and neurointensive care. Lancet Neurol (2011) 10:372–82 10.1016/S1474-4422(11)70016-3 21435600 PMC3757153

[19] Hope PL Reynolds EO Investigation of cerebral energy metabolism in newborn infants by phosphorus nuclear magnetic resonance spectroscopy. Clin Perinatol (1985) 12:261–75 3978989

[20] Azzopardi DV Strohm B Edwards AD Dyet L Halliday HL Juszczak E Moderate hypothermia to treat perinatal asphyxial encephalopathy. N Engl J Med (2009) 361:1349–58 10.1056/NEJMoa0900854 19797281

[21] Gluckman PD Wyatt JS Azzopardi D Ballard R Edwards AD Ferriero DM Selective head cooling with mild systemic hypothermia after neonatal encephalopathy: multicentre randomised trial. Lancet (2005) 365:663–70 10.1016/S0140-6736(05)17946-X 15721471

[22] Shankaran S Laptook AR Ehrenkranz RA Tyson JE McDonald SA Donovan EF Whole-body hypothermia for neonates with hypoxic-ischemic encephalopathy. N Engl J Med (2005) 353:1574–84 10.1056/NEJMcps050929 16221780

[23] Rutherford MA Azzopardi D Whitelaw A Cowan F Renowden S Edwards AD Mild hypothermia and the distribution of cerebral lesions in neonates with hypoxic-ischemic encephalopathy. Pediatrics (2005) 116:1001–6 10.1542/peds.2005-0328 16199715

[24] Stroke Therapy Academic Industry Roundtable (STAIR). Recommendations for standards regarding preclinical neuroprotective and restorative drug development. Stroke (1999) 30:2752–8 10.1161/01.STR.30.12.2752 10583007

[25] Stroke Therapy Academic Industry Roundtable II (STAIR-II). Recommendations for clinical trial evaluation of acute stroke therapies. Stroke (2001) 32:1598–606 10.1161/01.STR.32.7.1598 11441207

[26] Feuerstein GZ Zaleska MM Krams M Wang X Day M Rutkowski JL Missing steps in the STAIR case: a translational medicine perspective on the development of NXY-059 for treatment of acute ischemic stroke. J Cereb Blood Flow Metab (2008) 28:217–9 10.1038/sj.jcbfm.9600516 17579658

[27] Fisher M Feuerstein G Howells DW Hurn PD Kent TA Savitz SI Update of the stroke therapy academic industry roundtable preclinical recommendations. Stroke (2009) 40:2244–50 10.1161/STROKEAHA.108.541128 19246690 PMC2888275

[28] Chopp M Steinberg GK Kondziolka D Lu M Bliss TM Li Y Who’s in favor of translational cell therapy for stroke: STEPS forward please? Cell Transplant (2009) 18:691–3 10.3727/096368909X470883 19796499 PMC3962837

[29] Borlongan CV Chopp M Steinberg GK Bliss TM Li Y Lu M Potential of stem/progenitor cells in treating stroke: the missing steps in translating cell therapy from laboratory to clinic. Regen Med (2008) 3:249–50 10.2217/17460751.3.3.249 18462048 PMC7000104

[30] Borlongan CV Cell therapy for stroke: remaining issues to address before embarking on clinical trials. Stroke (2009) 40:S146–8 10.1161/STROKEAHA.108.533091 19064801 PMC4810678

[31] Wechsler L Steindler D Borlongan C Chopp M Savitz S Deans R Stem cell therapies as an emerging paradigm in stroke (STEPS): bridging basic and clinical science for cellular and neurogenic factor therapy in treating stroke. Stroke (2009) 40:510–5 10.1161/STROKEAHA.108.526863 19095993

[32] Borlongan CV Weiss MD Baby STEPS: a giant leap for cell therapy in neonatal brain injury. Pediatr Res (2011) 70:3–9 10.1038/pr.2011.228 21659957 PMC3117246

[33] Northington FJ Brief update on animal models of hypoxic-ischemic encephalopathy and neonatal stroke. ILAR J (2006) 47:32–8 10.1093/ilar.47.1.32 16391429

[34] Vannucci RC Vannucci SJ Perinatal hypoxic-ischemic brain damage: evolution of an animal model. Dev Neurosci (2005) 27:81–6 10.1159/000085978 16046840

[35] Ditelberg JS Sheldon RA Epstein CJ Ferriero DM Brain injury after perinatal hypoxia-ischemia is exacerbated in copper/zinc superoxide dismutase transgenic mice. Pediatr Res (1996) 39:204–8 10.1203/00006450-199602000-00003 8825788

[36] Sheldon RA Sedik C Ferriero DM Strain-related brain injury in neonatal mice subjected to hypoxia-ischemia. Brain Res (1998) 810:114–22 10.1016/S0006-8993(98)00892-0 9813271

[37] Fullerton HJ Ditelberg JS Chen SF Sarco DP Chan PH Epstein CJ Copper/zinc superoxide dismutase transgenic brain accumulates hydrogen peroxide after perinatal hypoxia ischemia. Ann Neurol (1998) 44:357–64 10.1002/ana.410440311 9749602

[38] Graham EM Sheldon RA Flock DL Ferriero DM Martin LJ O’Riordan DP Neonatal mice lacking functional Fas death receptors are resistant to hypoxic-ischemic brain injury. Neurobiol Dis (2004) 17:89–98 10.1016/j.nbd.2004.05.007 15350969

[39] Hagberg H Wilson MA Matsushita H Zhu C Lange M Gustavsson M PARP-1 gene disruption in mice preferentially protects males from perinatal brain injury. J Neurochem (2004) 90:1068–75 10.1111/j.1471-4159.2004.02547.x 15312162

[40] Yoles E Zarchin N Mayevsky A Effects of age on the metabolic, ionic and electrical response to anoxia in the newborn dog brain in vivo. J Basic Clin Physyol Pharmacol (1991) 2:297–313 10.1515/JBCPP.1991.2.4.297 1822145

[41] McQuillen PS Sheldon RA Shatz CJ Ferriero DM Selective vulnerability of subplate neurons after early neonatal hypoxia-ischemia. J Neurosci (2003) 23:3308–15 12716938 10.1523/JNEUROSCI.23-08-03308.2003 PMC6742293

[42] Wang S Wu EX Cai K Lau HF Cheung PT Khong PL Mild hypoxic-ischemic injury in the neonatal rat brain: longitudinal evaluation of white matter using diffusion tensor MR imaging. AJNR Am J Neuroradiol (2009) 30:1907–13 10.3174/ajnr.A1697 19749219 PMC7051303

[43] Huang Z Liu J Cheung PY Chen C Long-term cognitive impairment and myelination deficiency in a rat model of perinatal hypoxic-ischemic brain injury. Brain Res (2009) 1301:100–9 10.1016/j.brainres.2009.09.006 19747899

[44] Wang S Wu EX Tam CN Lau HF Cheung PT Khong PL Characterization of white matter injury in a hypoxic-ischemic neonatal rat model by diffusion tensor MRI. Stroke (2008) 39:2348–53 10.1161/STROKEAHA.107.509927 18535275

[45] Chang YC Huang CC Hung PL Huang HM Rolipram, a phosphodiesterase type IV inhibitor, exacerbates periventricular white matter lesions in rat pups. Pediatr Res (2008) 64:234–9 10.1203/PDR.0b013e31817cfc87 18437099

[46] Wen TC Rogido M Peng H Genetta T Moore J Sola A Gender differences in long-term beneficial effects of erythropoietin given after neonatal stroke in postnatal day-7 rats. Neuroscience (2006) 139:803–11 10.1016/j.neuroscience.2006.02.057 16581190

[47] Hurn PD Vannucci SJ Hagberg H Adult or perinatal brain injury: does sex matter? Stroke (2005) 36:193–5 10.1161/01.STR.0000153064.41332.f6 15625289

[48] Edwards RG Stem cells today: B1. Bone marrow stem cells. Reprod Biomed Online (2004) 9:541–83 10.1016/S1472-6483(10)61639-2 15588475

[49] Dewing P Shi T Horvath S Vilain E Sexually dimorphic gene expression in mouse brain precedes gonadal differentiation. Mol Brain Res (2003) 118:82–90 10.1016/S0169-328X(03)00339-5 14559357

[50] Carruth LL Reisert I Arnold AP Sex chromosome genes directly affect brain sexual differentiation. Nat Neurosci (2002) 5:933–4 10.1038/nn922 12244322

[51] Ifudu O Uribarri J Rajwani I Vlacich V Reydel K Delosreyes G Gender modulates responsiveness to recombinant erythropoietin. Am J Kidney Dis (2001) 38:518–22 10.1053/ajkd.2001.26842 11532683

[52] Zeng SM Yankowitz J Widness JA Strauss RG Etiology of differences in hematocrit between males and females: sequence-based polymorphisms in erythropoietin and its receptor. J Gend Specif Med (2001) 4:35–40 11324238

[53] Guo TL Germolec DR Musgrove DL Delclos KB Newbold RR Weis C Myelotoxicity in genistein-, nonylphenol-, methoxychlor-, vinclozolin- or ethinyl estradiol-exposed F1 generations of Sprague-Dawley rats following developmental and adult exposures. Toxicology (2005) 211:207–19 10.1016/j.tox.2005.03.008 15925024

[54] Pequignot JM Spielvogel H Caceres E Rodriguez A Sempore B Pequignot J Influence of gender and endogenous sex steroids on catecholaminergic structures involved in physiological adaptation to hypoxia. Pflugers Arch (1997) 433:580–6 10.1007/s004240050317 9049142

[55] Raju TN Some animal models for the study of perinatal asphyxia. Biol Neonate (1992) 62:202–14 10.1159/000243873 1420619

[56] Bjorkman ST Miller SM Rose SE Burke C Colditz PB Seizures are associated with brain injury severity in a neonatal model of hypoxia-ischemia. Neuroscience (2010) 166:157–67 10.1016/j.neuroscience.2009.11.067 20006975

[57] Zhang D Hathi M Yang ZJ Ding H Koehler R Thakor N Hypoxic-ischemic brain injury in neonatal piglets with different histological outcomes: an amplitude-integrated EEG study. Conf Proc IEEE Eng Med Biol Soc (2009) 2009:1127–30 10.1109/IEMBS.2009.5333439 19963989

[58] Yager JY Ashwal S Animal models of perinatal hypoxic-ischemic brain damage. Pediatr Neurol (2009) 40:156–67 10.1016/j.pediatrneurol.2008.10.025 19218028

[59] Tai WC Burke KA Dominguez JF Gundamraj L Turman JE Jr Growth deficits in a postnatal day 3 rat model of hypoxic-ischemic brain injury. Behav Brain Res (2009) 202:40–9 10.1016/j.bbr.2009.03.043 19447279

[60] Derrick M Luo NL Bregman JC Jilling T Ji X Fisher K Preterm fetal hypoxia-ischemia causes hypertonia and motor deficits in the neonatal rabbit: a model for human cerebral palsy? J Neurosci (2004) 24:24–34 10.1523/JNEUROSCI.2816-03.2004 14715934 PMC6729589

[61] Laptook AR Corbett RJ Burns D Sterett R Neonatal ischemic neuroprotection by modest hypothermia is associated with attenuated brain acidosis. Stroke (1995) 26:1240–6 10.1161/01.STR.26.7.1240 7604422

[62] Iwata O Iwata S Thornton JS De Vita E Bainbridge A Herbert L “Therapeutic time window” duration decreases with increasing severity of cerebral hypoxia-ischaemia under normothermia and delayed hypothermia in newborn piglets. Brain Res (2007) 1154:173–80 10.1016/j.brainres.2007.03.083 17475224

[63] Hess DC Borlongan CV Stem cells and neurological diseases. Cell Prolif (2008) 41(Suppl 1):94–114 10.1111/j.1365-2184.2008.00486.x 18181951 PMC6496373

[64] Hess DC Borlongan CV Cell-based therapy in ischemic stroke. Expert Rev Neurother (2008) 8:1193–201 10.1586/14737175.8.8.1193 18671663 PMC2753686

[65] Van Velthoven CTJ Kavelaars A van Bel F Heijnen CJ Repeated mesenchymal stem cell treatment after neonatal hypoxia-ischemia has distinct effects on formation and maturation of new neurons and oligodendrocytes leading to restoration of damage, corticospinal motor tract activity, and sensorimotor function. J Neurosci (2010) 30:9603–11 10.1523/JNEUROSCI.1835-10.2010 20631189 PMC6632441

[66] Borlongan CV Stahl CE Cameron DF Saporta S Freeman TB Cahill DW CNS immunological modulation of neural graft rejection and survival. Neurol Res (1996) 18:297–304 8875445 10.1080/01616412.1996.11740425

[67] Borlongan CV Koutouzis TK Jorden JR Martinez R Rodriguez AI Poulos SG Neural transplantation as an experimental treatment modality for cerebral ischemia. Neurosci Biobehav Rev (1997) 21:79–90 10.1016/0149-7634(95)00063-1 8994211

[68] Borlongan CV Tajima Y Trojanowski JQ Lee VM Sanberg PR Transplantation of cryopreserved human embryonal carcinoma-derived neurons (NT2N cells) promotes functional recovery in ischemic rats. Exp Neurol (1998) 149:310–21 10.1006/exnr.1997.6730 9500961

[69] Kondziolka D Wechsler L Goldstein S Meltzer C Thulborn KR Gebel J Transplantation of cultured human neuronal cells for patients with stroke. Neurology (2000) 55:565–9 10.1212/WNL.55.4.565 10953194

[70] Nelson PT Kondziolka D Wechsler L Goldstein S Gebel J DeCesare S Clonal human (hNT) neuron grafts for stroke therapy: neuropathology in a patient 27 months after implantation. Am J Pathol (2002) 160:1201–6 10.1016/S0002-9440(10)62546-1 11943704 PMC1867232

[71] Borlongan CV Lind JG Dillon-Carter O Yu G Hadman M Cheng C Bone marrow grafts restore cerebral blood flow and blood brain barrier in stroke rats. Brain Res (2004) 1010:108–16 10.1016/j.brainres.2004.02.072 15126123

[72] Newman MB Misiuta I Willing AE Zigova T Karl RC Borlongan CV Tumorigenicity issues of embryonic carcinoma-derived stem cells: relevance to surgical trials using NT2 and hNT neural cells. Stem Cells Dev (2005) 14:29–43 10.1089/scd.2005.14.29 15725742

[73] Hara K Matsukawa N Yasuhara T Xu L Yu G Maki M Transplantation of post-mitotic human neuroteratocarcinoma-overexpressing Nurr1 cells provides therapeutic benefits in experimental stroke: in vitro evidence of expedited neuronal differentiation and GDNF secretion. J Neurosci Res (2007) 85:1240–51 10.1002/jnr.21234 17335085

[74] Hara K Yasuhara T Maki M Matsukawa N Masuda T Yu SJ Neural progenitor NT2N cell lines from teratocarcinoma for transplantation therapy in stroke. Prog Neurobiol (2008) 85:318–34 10.1016/j.pneurobio.2008.04.005 18514379

[75] Yu G Fournier C Hess DC Borlongan CV Transplantation of carotid body cells in the treatment of neurological disorders. Neurosci Biobehav Rev (2005) 28:803–10 10.1016/j.neubiorev.2004.09.011 15642622

[76] Sun J Allison J McLaughlin C Sledge L Waters-Pick B Wease S Differences in quality between privately and publicly banked umbilical cord blood units: a pilot study of autologous cord blood infusion in children with acquired neurologic disorders. Transfusion (2010) 50:1980–7 10.1111/j.1537-2995.2010.02720.x 20546200 PMC3816574

[77] Portmann-Lanz CB Schoeberlein A Huber A Sager R Malek A Holzgreve W Placental mesenchymal stem cells as potential autologous graft for pre- and perinatal neuroregeneration. Am J Obstet Gynecol (2006) 194:664–73 10.1016/j.ajog.2006.01.101 16522395

[78] Nishino H Borlongan CV Restoration of function by neural transplantation in the ischemic brain. Prog Brain Res (2000) 127:461–76 10.1016/S0079-6123(00)27022-2 11142041

[79] Matsukawa N Yasuhara T Hara K Xu L Maki M Yu G Therapeutic targets and limits of minocycline neuroprotection in experimental ischemic stroke. BMC Neurosci (2009) 10:126 10.1186/1471-2202-10-126 19807907 PMC2762982

[80] Robertson CM Finer NN Long-term follow-up of term neonates with perinatal asphyxia. Clin Perinatol (1993) 20:483–500 7689432

[81] Lauterbach MD Raz S Sander CJ Neonatal hypoxic risk in preterm birth infants: the influence of sex and severity of respiratory distress on cognitive recovery. Neuropsychology (2001) 15:411–20 10.1037/0894-4105.15.3.411 11499996

[82] Espy KA Senn TE Charak DA Tyler J Wiebe SA Perinatal pH and neuropsychological outcomes at age 3 years in children born preterm: an exploratory study. Dev Neuropsychol (2007) 32:669–82 10.1080/87565640701376003 17931124

[83] Kaandorp JJ Benders MJ Rademaker CM Torrance HL Oudjik MA Haan TR Antenatal allopurinol for reduction of birth asphyxia induced brain damage (ALLO-trial); a randomized double blind placebo controlled multicenter study. BMC Pregnancy Childbirth (2010) 10:8 10.1186/1471-2393-10-8 20167117 PMC2834613

[84] Hobbs C Thoresen M Tucker A Aquilina K Chakkarapani E Dingley J Xenon and hypothermia combine additively, offering long-term functional and histopathologic neuroprotection after neonatal hypoxia/ischemia. Stroke (2008) 39:1307–13 10.1161/STROKEAHA.107.499822 18309163

[85] Carroll JE Borlongan CV Adult stem cell therapy for acute brain injury in children. CNS Neurol Disord Drug Targets (2008) 7:361–9 10.2174/187152708786441812 18991664

[86] Kim CT Han J Kim H Pediatric stroke recovery: a descriptive analysis. Arch Phys Med Rehabil (2009) 90:657–62 10.1016/j.apmr.2008.10.016 19345783

[87] Yasuhara T Hara K Maki M Mays RW Hess DC Carroll JE Intravenous grafts recapitulate the neurorestoration afforded by intracerebrally delivered multipotent adult progenitor cells in neonatal hypoxic-ischemic rats. J Cereb Blood Flow Metab (2008) 28:1804–10 10.1038/jcbfm.2008.68 18594556 PMC2587070

[88] Yasuhara T Borlongan CV Date I Ex vivo gene therapy: transplantation of neurotrophic factor-secreting cells for cerebral ischemia. Front Biosci (2006) 11:760–75 10.2741/1834 16146768

[89] Yasuhara T Matsukawa N Yu G Xu L Mays RW Kovach J Behavioral and histological characterization of intrahippocampal grafts of human bone marrow-derived multipotent progenitor cells in neonatal rats with hypoxic-ischemic injury. Cell Transplant (2006) 15:231–8 10.3727/000000006783982034 16719058

[90] Parolini O Alviano F Bergwerf I Boraschi D De Bari C De Waele P Toward cell therapy using placenta-derived cells: disease mechanisms, cell biology, preclinical studies, and regulatory aspects at the round table. Stem Cells Dev (2010) 19:143–54 10.1089/scd.2009.0404 19947828

[91] Ashwal S Obenaus A Snyder EY Neuroimaging as a basis for rational stem cell therapy. Pediatr Neurol (2009) 40:227–36 10.1016/j.pediatrneurol.2008.09.025 19218036

[92] Chau V Poskitt KJ Miller SP Advanced neuroimaging techniques for the term newborn with encephalopathy. Pediatr Neurol (2009) 40:181–8 10.1016/j.pediatrneurol.2008.09.012 19218031

[93] Agrawal N Johnston SC Wu YW Sidney S Fullerton HJ Imaging data reveal a higher pediatric stroke incidence than prior US estimates. Stroke (2009) 40:3415–21 10.1161/STROKEAHA.109.564633 19762687 PMC3387270

[94] Hoehn M Wiedermann D Justicia C Ramos-Cabrer P Kruttwig K Farr T Cell tracking using magnetic resonance imaging. J Physiol (2007) 584:25–30 10.1113/jphysiol.2007.139451 17690140 PMC2277052

[95] Modo M Mellodew K Cash D Fraser SE Meade TJ Price J Mapping transplanted stem cell migration after a stroke: a serial, in vivo magnetic resonance imaging study. Neuroimage (2004) 21:311–7 10.1016/j.neuroimage.2003.08.030 14741669

[96] Jendelova P Herynek V Urdzikova L Glogarova K Kroupova J Andersson B Magnetic resonance tracking of transplanted bone marrow and embryonic stem cells labeled by iron oxide nanoparticles in rat brain and spinal cord. J Neurosci Res (2004) 76:232–43 10.1002/jnr.20041 15048921

[97] Shyu WC Chen CP Lin SZ Lee YJ Li H Efficient tracking of non-iron-labeled mesenchymal stem cells with serial MRI in chronic stroke rats. Stroke (2007) 38:367–74 10.1161/01.STR.0000254463.24655.14 17194887

[98] Song M Kim Y Ryu S Song I Kim SU Yoon BW MRI tracking of intravenously transplanted human neural stem cells in rat focal ischemia model. Neurosci Res (2009) 64:235–9 10.1016/j.neures.2009.03.006 19428705

[99] Daadi MM Li Z Arac A Grueter BA Sofilos M Malenka RC Molecular and magnetic resonance imaging of human embryonic stem cell-derived neural stem cell grafts in ischemic rat brain. Mol Ther (2009) 17:1282–91 10.1038/mt.2009.104 19436269 PMC2835224

[100] Lee ES Chan J Shuter B Tan LG Chong MS Ramachandra DL Microgel iron oxide nanoparticles for tracking human fetal mesenchymal stem cells through magnetic resonance imaging. Stem Cells (2009) 27:1921–31 10.1002/stem.112 19544438

[101] Tajiri N Kaneko Y Shinozuka K Ishikawa H Yankee E McGrogan M Stem cell recruitment of newly formed host cells via a successful seduction? Filling the gap between neurogenic niche and injured brain site. PLoS One (2013) 8:e74857 10.1371/journal.pone.0074857 24023965 PMC3762783

[102] Sharma A Sane H Gokulchandran N Badhe P Paranjape K Paranjape A Stem cell therapy for cerebral palsy – a novel option. In: Svaraka E, editor. Cerebral Palsy – Challenges for the Future. InTechOpen (2014). Available from: http://www.intechopen.com/books/cerebral-palsy-challenges-for-the-future/stem-cell-therapy-for-cerebral-palsy-a-novel-option

[103] ClinicalTrials.gov. Available from: http://clinicaltrials.gov/ct2/results?term=stem+cells+AND+cerebral+palsy&Search=Search (2014)

[104] Liao Y Cotten M Tan S Kurtzberg J Cairo MS Rescuing the neonatal brain from hypoxic injury with autologous cord blood. Bone Marrow Transplant (2012) 48:890–900 10.1038/bmt.2012.169 22964590

[105] Li M Yu A Zhang F Dai G Cheng H Wang X Treatment of one case of cerebral palsy combined with posterior visual pathway injury using autologous bone marrow mesenchymal stem cells. J Transl Med (2012) 10:100 10.1186/1479-5876-10-100 22607263 PMC3479002

[106] Jordan LC Rafay MF Smith SE Askalan R Zamel KM deVeber G Antithrombotic treatment in neonatal cerebral sinovenous thrombosis: results of the International Pediatric Stroke Study. J Pediatr (2010) 156:704–10 10.1016/j.jpeds.2009.11.061 20149389 PMC2854210

[107] Grunwald IQ Walter S Shamdeen MG Dautermann A Roth C Haass A New mechanical recanalization devices – the future in pediatric stroke treatment? J Invasive Cardiol (2010) 22:63–6 20124590

[108] Normann S De Veber G Fobker M Langer C Kenet G Bernanrd TJ Role of endogenous testosterone concentration in pediatric stroke. Ann Neurol (2009) 66:754–8 10.1002/ana.21840 20033984

[109] Kenet G Lutkhoff LK Albisetti M Bernanrd T Bonduel M Brandao L Impact of thrombophilia on risk of arterial ischemic stroke or cerebral sinovenous thrombosis in neonates and children: a systematic review and meta-analysis of observational studies. Circulation (2010) 121:1838–47 10.1161/CIRCULATIONAHA.109.913673 20385928

[110] Ganesan V Pediatric stroke guidelines: where will these take future research and treatment options for childhood stroke? Expert Rev Neurother (2009) 9:639–48 10.1586/ern.09.14 19402775

[111] Amlie-Lefond C Bernard TJ Sebire G Friedman NR Heyer GL Lerner NB Predictors of cerebral arteriopathy in children with arterial ischemic stroke: results of the International Pediatric Stroke Study. Circulation (2009) 119:1417–23 10.1161/CIRCULATIONAHA.108.806307 19255344 PMC4205969

[112] Berman DR Liu Y Barks J Mozurkewich E Treatment with docosahexaenoic acid after hypoxia-ischemia improves forepaw placing in a rat model of perinatal hypoxia-ischemia. Am J Obstet Gynecol (2010) 203:381–5 10.1016/j.ajog.2010.06.017 20691409 PMC2947568

[113] Zhou Y Fathali N Lekic T Ostrowski RP Chen C Martin RD Remote limb ischemic postconditioning protects against neonatal hypoxic-ischemic brain injury in rat pups by the opioid receptor/Akt pathway. Stroke (2011) 42:439–44 10.1161/STROKEAHA.110.592162 21183744 PMC3703505

[114] Shankaran S Laptook A Wright LL Ehrenkranz RA Donovan EF Fanaroff AA Whole-body hypothermia for neonatal encephalopathy: animal observations as a basis for a randomized, controlled pilot study in term infants. Pediatrics (2002) 110:377–85 10.1542/peds.110.2.377 12165594

[115] Lei B Tan X Cai H Xu Q Guo Q Effect of moderate hypothermia on lipid peroxidation in canine brain tissue after cardiac arrest and resuscitation. Stroke (1994) 25:147–52 10.1161/01.STR.25.1.147 8266363

[116] Thoresen M Penrice J Lorek A Cady EB Wylezinska M Kirkbride V Mild hypothermia after severe transient hypoxia-ischemia ameliorates delayed cerebral energy failure in the newborn piglet. Pediatr Res (1995) 37:667–70 10.1203/00006450-199505000-00019 7603788

[117] Battin MR Dezoete JA Gunn TR Gluckman PD Gunn AJ Neurodevelopmental outcome of infants treated with head cooling and mild hypothermia after perinatal asphyxia. Pediatrics (2001) 107:480–4 10.1542/peds.107.3.480 11230586

[118] Battin MR Penrice J Gunn TR Gunn AJ Treatment of term infants with head cooling and mild systemic hypothermia (35.0 degrees C and 34.5 degrees C) after perinatal asphyxia. Pediatrics (2003) 111:244–51 10.1542/peds.111.2.244 12563046

[119] Bergstedt K Hu BR Wieloch T Postischaemic changes in protein synthesis in the rat brain: effects of hypothermia. Exp Brain Res (1993) 95:91–9 10.1007/BF00229658 8405256

[120] Tagin MA Woolcott CG Vincer MJ Whyte RK Stinson DA Hypothermia for neonatal hypoxic ischemic encephalopathy: an updated systematic review and meta-analysis. Arch Pediatr Adolesc Med (2012) 166:558–66 10.1001/archpediatrics.2011.1772 22312166

[121] Pavel Gonzalez-Ibarra F Varon J Lopez-Meza EG Therapeutic hypothermia: critical review of the molecular mechanism of action. Front Neurol (2011) 2:4 10.3389/fneur.2011.00004 21331282 PMC3035015

[122] Deng W Aimone JB Gage FH New neurons and new memories: how does adult hippocampal neurogenesis affect learning and memory? Nat Rev Neurosci (2010) 11:339–50 10.1038/nrn2822 20354534 PMC2886712

[123] Zlokovic BV Neurovascular pathways to neurodegeneration in Alzheimer’s disease and other disorders. Nat Rev Neurosci (2011) 12:723–38 10.1038/nrn3114 22048062 PMC4036520

[124] Yenari M Kitagawa K Lyden P Perez-Pinzon M Metabolic downregulation: a key to successful neuroprotection? Stroke (2008) 39:2910–7 10.1161/STROKEAHA.108.514471 18658035 PMC2597567

[125] Robertson NJ Nakakeeto M Hagmann C Cowan FM Acolet D Iwata O Therapeutic hypothermia for birth asphyxia in low-resource settings: a pilot randomised controlled trial. Lancet (2008) 372:801–3 10.1016/S0140-6736(08)61329-X 18774411

[126] Glass HC Nash KB Bonifacio SL Barkovich AJ Ferriero DM Sullivan JE Seizures and magnetic resonance imaging-detected brain injury in newborns cooled for hypoxic-ischemic encephalopathy. J Pediatr (2011) 159: 731–5.e1 10.1016/j.jpeds.2011.07.015 21839470 PMC3193544

[127] Higgins RD Raju T Edwards AD Azzopardi DV Bose CL Clark RH Hypothermia and other treatment options for neonatal encephalopathy: an executive summary of the Eunice Kennedy Shriver NICHD workshop. J Pediatr (2011) 159(851–858):e851 10.1016/j.jpeds.2011.08.004 PMC3263823 21875719

[128] Jatana M Singh I Singh AK Jenkins D Combination of systemic hypothermia and N-acetylcysteine attenuates hypoxic-ischemic brain injury in neonatal rats. Pediatr Res (2006) 59:684–9 10.1203/01.pdr.0000215045.91122.44 16627882

[129] Jenkins DD Chang E Singh I Neuroprotective interventions: is it too late? J Child Neurol (2009) 24:1212–9 10.1177/0883073809338412 19745093 PMC3674502

[130] Wu YW Bauer LA Ballard RA Ferriero DM Glidden DV Mayock DE Erythropoietin for neuroprotection in neonatal encephalopathy: safety and pharmacokinetics. Pediatrics (2012) 130:683–91 10.1542/peds.2012-0498 23008465 PMC3457622

[131] Xiong T Qu Y Mu D Ferriero D Erythropoietin for neonatal brain injury: opportunity and challenge. Int J Dev Neurosci (2011) 29:583–91 10.1016/j.ijdevneu.2010.12.007 21277366

[132] Dickinson R Franks NP Bench-to-bedside review: molecular pharmacology and clinical use of inert gases in anesthesia and neuroprotection. Crit Care (2010) 14:229 10.1186/cc9051 20836899 PMC2945072

[133] Ferriero DM Neonatal brain injury. N Engl J Med (2004) 351:1985–95 10.1056/NEJMra041996 15525724

[134] Bonifacio SL Glass HC Peloquin S Ferriero DM A new neurological focus in neonatal intensive care. Nat Rev Neurol (2011) 7:485–94 10.1038/nrneurol.2011.119 21808297

[135] Borlongan CV Glover LE Tajiri N Kaneko Y Freeman TB The great migration of bone marrow-derived stem cells toward the ischemic brain: therapeutic implications for stroke and other neurological disorders. Prog Neurobiol (2011) 95:213–28 10.1016/j.pneurobio.2011.08.005 21903148 PMC3185169

[136] Borlongan CV Kaneko Y Maki M Yu SJ Ali M Allickson JG Menstrual blood cells display stem cell-like phenotypic markers and exert neuroprotection following transplantation in experimental stroke. Stem Cells Dev (2010) 19:439–52 10.1089/scd.2009.0340 19860544 PMC3158424

[137] Ozawa K Sato K Oh I Ozaki K Uchibori R Obara Y Cell and gene therapy using mesenchymal stem cells (MSCs). J Autoimmun (2008) 30:121–7 10.1016/j.jaut.2007.12.008 18249090

[138] Gunn AJ Gunn TR The “pharmacology” of neuronal rescue with cerebral hypothermia. Early Hum Dev (1998) 53:19–35 10.1016/S0378-3782(98)00033-4 10193924

[139] Iadecola C Anrather J Stroke research at a crossroad: asking the brain for directions. Nat Neurosci (2011) 14:1363–8 10.1038/nn.2953 22030546 PMC3633153

[140] Edwards AD Brocklehurst P Gunn AJ Halliday H Juszczak E Levene M Neurological outcomes at 18 months of age after moderate hypothermia for perinatal hypoxic ischaemic encephalopathy: synthesis and meta-analysis of trial data. BMJ (2010) 340:c363 10.1136/bmj.c363 20144981 PMC2819259

[141] Shalak L Perlman JM Hypoxic-ischemic brain injury in the term infant-current concepts. Early Hum Dev (2004) 80:125–41 10.1016/j.earlhumdev.2004.06.003 15500993

[142] Goñi de Cerio F Lara-Celador I Alvarez A Hilario E Neuroprotective therapies after perinatal hypoxic-ischemic brain injury. Brain Sci (2013) 3:191–214 10.3390/brainsci3010191 24961314 PMC4061821

[143] Sims B Neuroprotective strategies in neonatal brain injury. Pediat Therapeut (2012) 2:e117 10.4172/2161-0665.1000e117

[144] Carrascosa-Romero MC De Cabo-de la Vega C Neuroprotection in perinatal hypoxic-ischemic encephalopathy – pharmacologic combination therapy. In: Švraka E, editor. Cerebral Palsy – Challenges for the Future. Rijeka, Croatia: InTech (2014). p. 123–92

[145] Zhuang L Yang T Zhao H Fidalgo AR Vizcaychipi M Sanders RD The protective profile of argon, helium, and xenon in a model of neonatal asphycia in rats. Crit Care Med (2012) 40:1724–30 10.1097/CCM.0b013e3182452164 22610177

[146] Boutin H Dauphin F Mackenzie ET Jauzac P Differential time-course decreases in nonselective, mu-, delta-, and kappa-opioid receptors after focal cerebral ischemia in mice. Stroke (1999) 30:1271–8 10.1161/01.STR.30.6.1271 10356111

[147] Kevelaitis E Peynet J Mouas C Launay JM Menasche P Opening of potassium channels: the common cardioprotective link between preconditioning and natural hibernation? Circulation (1999) 99:3079–85 10.1161/01.CIR.99.23.3079 10368128

[148] Tsai SY Lee CT Hayashi T Freed WJ Su TP Delta opioid peptide DADLE and naltrexone cause cell cycle arrest and differentiation in a CNS neural progenitor cell line. Synapse (2010) 64:267–73 10.1002/syn.20727 19953654 PMC3155725

[149] Borlongan CV Su TP Wang Y Treatment with delta opioid peptide enhances in vitro and in vivo survival of rat dopaminergic neurons. Neuroreport (2000) 11:923–6 10.1097/00001756-200004070-00005 10790856

[150] Kaneko Y Tajiri N Su TP Wang Y Borlongan CV Combination treatment of hypothermia and mesenchymal stromal cells amplifies neuroprotection in primary rat neurons exposed to hypoxic-ischemic-like injury in vitro: role of the opioid system. PLoS One (2012) 7:e47583 10.1371/journal.pone.0047583 23077646 PMC3471862

[151] Young MN Hollenbeck RD Pollock JS McPherson JA Fredi JL Piana RM Effectiveness of mild therapeutic hypothermia following cardiac arrest in adult patients with congenital heart disease. Am J Cardiol (2014) 114:128–30 10.1016/j.amjcard.2014.04.012 24819894

[152] Crossley S Reid J McLatchie R Hayton J Clark C MacDougal M A systematic review of therapeutic hypothermia for adult patients following traumatic brain injury. Crit Care (2014) 18:R75 10.1186/cc13835 24742169 PMC4056614

[153] Batchelor PE Skeers P Antonic A Wills TE Howells DW Macleod MR Systematic review and meta-analysis of therapeutic hypothermia in animal models of spinal cord injury. PLoS One (2013) 8:e71317 10.1371/journal.pone.0071317 23951131 PMC3739756

[154] Tang XN Liu L Koike MA Yenari MA Mild hypothermia reduces tissue plasminogen activator-related hemorrhage and blood brain barrier disruption after experimental stroke. Ther Hypothermia Temp Manag (2013) 3:74–83 10.1089/ther.2013.0010 23781399 PMC3684213

[155] Marchetto M Gage FH Your brain under the microscope: the promise of stem cells. Cerebrum (2014) 2014:1 25009691 PMC4087191

[156] Barker RA Jain M Armstrong RJE Caldwell MA Stem cells and neurological disorders. J Neurol Neurosurg Psychiatry (2003) 74:553–7 10.1136/jnnp.74.5.553 12700287 PMC1738434

[157] Borlongan CV Lind JG Dillon-Carter O Yu G Hadman M Cheng C Intracerebral xenografts of mouse bone marrow cells in adult rats facilitate restoration of cerebral blood flow and blood-brain barrier. Brain Res (2004) 1009:26–33 10.1016/j.brainres.2004.02.050 15120580

[158] Borlongan CV Hadman M Sanberg CD Sanberg PR Central nervous system entry of peripherally injected umbilical cord blood cells is not required for neuroprotection in stroke. Stroke (2004) 35:2385–9 10.1161/01.STR.0000141680.49960.d7 15345799

[159] Borlongan CV Hida H Nishino H Early assessment of motor dysfunctions aids in successful occlusion of the middle cerebral artery. Neuroreport (1998) 9:3615–21 10.1097/00001756-199811160-00012 9858369

